# Intelligence, Correspondence, &c.

**Published:** 1835-07-01

**Authors:** 


### INTELLIGENCE, CORRESPONDENCE, &c.

In our advertising sheet will be found a notice of the new School of Anatomy, *Kinnerton Street*, contiguous to St. George's Hospital. This School, almost unequalled for the accommodations it offers to Pupils, will be opened in *October* next. Mr. Tatum, Mr. H. Johnson (the junior Editor of this Journal), and Mr. Henry Charles Johnson, are the Lecturers and Demonstrators. Gentlemen from the Country will find at this School every possible arrangement conducive to their health and comforts.

We have received the two cases of Prolapsus Ani, cured by excision, from Mr. James Edwards, of Forfar. The operations were performed according to the practice of the best metropolitan surgeons ; but we think the details of the cases are unnecessary.

From Mr. Groome, of Whitchurch, Salop, we have received the Drawings of a most extraordinary Case, of which but a very inadequate idea can be conveyed in words.

"The patient is a woman, 31 years of age, of a scrofulous diathesis—her health not so much impaired as might be expected, from the immense size of the tumour and long confinement. She first perceived it when 11 years old ; it very gradually increased until she arrived at her twenty-sixth year, in which year she had an illegitimate child ; she menstruated three times afterwards, the last time being in August, 1830. After that period, its growth was much more rapid, in 1832 the weight being so great, she could not be moved without great difficulty and pain ; she, therefore, took to her bed, and has not been able to leave it since. She informs me she perceives it larger every month, and that the increase has been greater the last three years than the whole former period. The circumference of the tumor, anteriorly, is 62 inches, longitude 36 inches.—Posterior view, in circumference, 76 inches.

Its origin is from the left hip and thigh ; indeed it and the leg are completely enveloped in the mass, to within three inches of the ankle. The spine is much distorted, but was not so until the tumor was of considerable magnitude ; it is sensitive in every part, the heat is greater than in any other part of the body."

We have received an apparatus for more correctly measuring minims, by Mr. R. Allsop, Operative Chemist, of Sloane Street. We have tried it, and it certainly appears to secure greater accuracy of measurement than the common minim-glass can pretend to.

Dr. Gilkrest, Deputy Inspector General of Hospitals at Gibraltar, has been recently elected a Corresponding Member of the Royal Academy of Medicine, at Paris.

(*Communicated by R. Montgomery Martin.*)

Medico-chirurgical Review.

A Set of the above Journal, 1 to 40, with the exception of a single Number (27), to be sold by Mr. Sanders, *Little Shire Lane*, for £3. 3*s*.

*In the press,*

Practical Observations on the Nature and Treatment of Nervous Diseases, with Remarks on the Efficacy of Strychnine in the more obstinate Cases. By G. Russell Mart, M.R.C.S. and late of His Majesty's hospital ship, Racoon.

